# Associations Between Sleep Duration and Activity of Daily Living Disability Among Older Adults in China: Cross-Sectional Study

**DOI:** 10.2196/65075

**Published:** 2025-03-05

**Authors:** Huimin Fan, Weijie Yu, Hongguo Rong, Xiaokun Geng

**Affiliations:** 1 Department of Neurology, Beijing Luhe Hospital, Capital Medical University, Beijing, China; 2 School of Traditional Chinese Medicine, Beijing University of Chinese Medicine, Beijing, China; 3 Institute for Excellence in Evidence-Based Chinese Medicine, Beijing University of Chinese Medicine, No. 11 Beisanhuan East Road, Chaoyang District, Beijing, 100029, China, 86 (10)64286757; 4 China-America Institute of Neuroscience, Beijing Luhe Hospital, Capital Medical University, Beijing, China; 5 Stroke Center, Department of Neurology, Beijing Luhe Hospital, Capital Medical University, No. 82 Xinhua SouthRoad, Tongzhou DistrictBeijing, 101149, China

**Keywords:** sleep, sleep duration, activities of daily living, CHARLS, survey, questionnaire, self-reported, gerontology, geriatric, older adult, elder, elderly, aging, ADL, physical function, physical functioning, well-being, association, correlation, China Health and Retirement Longitudinal Study

## Abstract

**Background:**

China has the largest elderly population globally; the growth rate of the aged tendency of the population was higher than that of Western countries. Given the distinctions in historical, ethnic, and economic status as well as socio-cultural background, Chinese adults had different sleep patterns compared with adults in other countries. Considering the heavy disease burden caused by activities of daily living (ADL) disability, we conducted a cross-sectional analysis using data from the China Health and Retirement Longitudinal Study (CHARLS) to test the hypothesis that individuals with short and longer sleep duration are more likely to have ADL disability.

**Objective:**

ADL disability is a common condition affecting the quality of life among older people. This study aimed to explore the associations between sleep duration and ADL disability among middle-aged and older adults in China.

**Methods:**

This cross-sectional study used data from 17,607 participants from the 2018 CHARLS (from 2018 to 2020), an ongoing representative survey of adults aged 45 years or older and their spouses. Self-reported sleep duration per night was obtained from face-to-face interviews. The ADL was measured using a 6-item summary assessed with an ADL scale that included eating, dressing, getting into or out of bed, bathing, using the toilet, and continence. Multiple generalized linear regression models—adjusted for age, sex, education, marital status, tobacco and alcohol use, depression, place of residence, sensory impairment, self-reported health status, life satisfaction, daytime napping, chronic disease condition, and sample weights—were used.

**Results:**

Data were analyzed from 17,607 participants, of whom 8375 (47.6%) were men. The mean (SD) age was 62.7 (10.0) years. Individuals with 4 hours or less (odds ratio [OR] 1.91, 95% CI 1.60‐2.27; *P*<.001), 5 hours (OR 1.33, 95% CI 1.09‐1.62; *P*=.006), 9 hours (OR 1.48, 95% CI 1.13‐1.93; *P*<.001), and 10 hours or more (OR 1.88, 95% CI 1.47‐2.14*; P*<.001) of sleep per night had a higher risk of ADL disability than those in the reference group (7 hours per night) after adjusting for several covariates. Restricted cubic splines analysis suggested a U-shaped association between sleep duration and ADL disability. When sleep duration fell below 7 hours, an increased sleep duration was associated with a significantly low risk of ADL disability, which was negatively correlated with sleep duration until it fell below 7 hours (OR 0.83, 95% CI 0.79‐0.87; *P*<.001). When sleep duration exceeded 7 hours, the risk of ADL disability would increase facing prolonged sleep duration (OR 1.19, 95% CI 1.12‐1.27; *P*<.001). ADL disability should be monitored in individuals with insufficient (≤4 or 5 hours per night) or excessive (9 or ≥10 hours per night) sleep duration.

**Conclusions:**

In this study, a U-shaped association between sleep duration and ADL disability was found. Future longitudinal studies are needed to establish temporality and examine the mechanisms of the associations between sleep duration and ADL disability.

## Introduction

The World Health Organization (WHO) survey of 2022 reported that 46.1% of adults aged 60 years and older are living with a disability globally [1]. Furthermore, according to the WHO projections, there will be 66 million older adults in China who live with a functional handicap by 2050 [1]. Activities of daily living (ADL) disability are one of the most common health problems in older adults, affecting about 1 in 6 older adults worldwide [2]. The prevalence of ADL disability increased with aging and had become a significant factor that increases the risk of mortality in older adults [3,4]. ADL disability seriously affected the quality of life and placed a high burden on care providers and the care system. Current research has focused on the prevention of, rehabilitation from, or management of ADL disability, with growing interest in the role of sleep patterns, such as sleep duration [5-7].

Sleep duration has become a significant public health issue [8], associated with the increasing risk of cardiovascular diseases, stroke, and mortality [9-11]. The WHO launched the ”World Mental Health Report—Transforming Health for All” on June 17, 2022 [12]. This report emphasizes that sleep loss is an extremely important risk factor for mental health issues. At present, the relationship between sleep duration and ADL disability has received much attention [13]. A longitudinal observational study suggested that short sleep duration during inpatient rehabilitation might be against greater functional ability at discharge among individuals with acute stroke [14]. The data from the National Health and Nutrition Examination Survey in the United States found that only short sleep duration was significantly associated with ADL disability [15]. The New Integrated Suburban Seniority Investigation Project study also reported that sleeping <6 hours/day was associated with a higher risk of incident disability among older Japanese people [16]. Although the above study did not find a relationship between long sleep and physical activity, the data from the English Longitudinal Study of Ageing cohort showed that both reduced and increased sleep duration were associated with limited agility and mobility disability [17]. A cross-sectional study in Taiwan also reported long sleep duration had a positive association with grip strength performance among older adults [18]. There is still no unified conclusion in the research on the relationship between sleep duration and ADL disability. So far, research on the relationship between sleep duration and ADL disability is well worth conducting.

China has the largest population of older adults globally; the growth rate of the aged tendency of the population was higher than that of Western countries [19,20]. Given the distinctions in historical, ethnic, and economic status as well as socio-cultural backgrounds, Chinese adults had different sleep patterns compared to those from other countries [21,22]. Considering the heavy disease burden caused by ADL disability, we conducted a cross-sectional analysis using data from the China Health and Retirement Longitudinal Study (CHARLS) to test the hypothesis that individuals with short and longer sleep duration are more likely to have ADL disability.

## Methods

### Ethical Considerations

The CHARLS protocol was approved by the Peking University Biomedical Ethics Review Board (IRB00001052-11015) [23,24]. This cross-sectional study adhered to the reporting guidelines stipulated by the Strengthening the Reporting of Observational Studies in Epidemiology (STROBE) reporting guideline [25]. According to the Common Rule (45 CFR part 46), this study was exempt from institutional review board approval and the requirement for informed patient consent because we did not use clinical data or involve human participants.

### Study Participants

This cross-sectional study used data from the 2018 survey of the CHARLS conducted from 2018 to 2020, a survey of residents aged 45 years or older in 28 provinces across China [26,27]. The national baseline survey of the CHARLS included 17,708 respondents in 150 counties or districts and 450 villages or urban communities throughout the country. Detailed methods of the CHARLS have been described previously [23]. We applied the latest CHARLS wave in 2018 to 19,816 individuals to investigate the relationship between sleep duration and ADL disability. In the current study, we formulated the inclusion criteria for the study subjects: age ≥45 years, respondents with complete information on sleep duration, the ADL scale, and other covariates. We excluded the following outliers: missing date of sample weights, birth date, sleep duration, and ADL scale.

### Sleep Duration

Similar to the English Longitudinal Study of Aging [28] in England, the CHARLS evaluated subjects’ sleep duration by utilizing the questions: “During the past month, how many hours of actual sleep did you get at night (average hours for one night)? (This might be shorter than the number of hours you spend in bed).” The response was an average sleep duration of typical usual weekdays or workdays within a month. Consistent with the previous CHARLS sleep study [29-32], we separated respondents into 7 sleep duration groups (≤4, 5, 6, 7, 8, 9, and ≥10 hours out of each evening) in the research. The CHARLS used this question to assess participants’ daytime napping: “In the past month, how long have you taken on average for a nap after lunch?” On the basis of 4 nap time groups considering the results [33,34], the participants were classified as nonnappers (0 min), short nappers (<30 minutes), moderate nappers (30‐90 minutes), and extended nappers (>90 minutes).

### ADL Disability

The CHARLS employed a 6-item summary derived from an ADL scale to quantify ADL utilizing a comprehensive series of inquiries that asked: “Are you experiencing any difficulties with performing any everyday activity due to physical, mental, emotional, or memory impairments, excluding any that you anticipate will persist for less than three months?” The daily tasks encompassed dressing, bathing or showering, eating, getting into or out of bed, using the toilet, and controlling urination and defecation. The response scale contained 4 options: (1) No, I don’t have any difficulty; (2) I have difficulty but can still do it; (3) Yes, I have difficulty and need help; and (4) I cannot do it. The ADL scale was extensively used in previous studies among Chinese older adults and has shown good reliability and validity [35,36]. Individuals classified as independent possessed the capability to perform all 6 activities without any difficulty, whereas those who indicated a need for assistance in any of the aforementioned tasks were considered to have an ADL disability [37,38].

### Covariates

The CHARLS collected information on sociodemographic characteristics and health-related factors using a structured questionnaire. Covariates that might confound the associations included age, sex, education (elementary school and below, secondary school, and college and above), marital status (married and others), residence (rural or urban), smoking and drinking status (never vs current), visual impairment, hearing impairment, self-reported general health status (ie, very good, good, fair, poor, and very poor), self-reported life satisfaction (ie, completely satisfied, very satisfied, somewhat satisfied, not very satisfied, or not at all satisfied), daytime napping, depression, chronic disease condition (ie, none, mild, and severe), and sample weights. Respondents were deemed to have visual or hearing impairment if they reported fair or poor vision (for either long-distance or near vision) or hearing difficulties. The respondents’ chronic disease status was documented based on the count of self-reported noncommunicable diseases, encompassing hypertension, diabetes, dyslipidemia, heart conditions, stroke, renal diseases, asthma, pulmonary disorders, arthritis, liver ailments, and gastric issues. As for sampling weights, the CHARLS constructed cross-sectional sample weights directly from the sampling probabilities for households and individuals, taking into account death and divorce. In the 2018 survey, the CHARLS provides two sets of cross-sectional household weights, one with corrections for nonresponse and one without. Our study adopted the sets of cross-sectional individual weights with corrections.

### Statistical Analysis

In the current study, comparisons of respondents’ general characteristics based on ADL disability status were conducted using analysis of variance (ANOVA) for numerical variables and ordinal *χ*^2^ tests for categorical variables. Numerical variables were presented as the mean (SD). Categorical variables were reported as numbers and percentages. Given the dichotomous nature of the ADL disability measure utilized in this study, we adopted multivariable generalized linear models with binomial family and log links to examine the associations between sleep duration and ADL disability; meanwhile, the odds ratios (ORs) and 95% CIs were reported. Restricted cubic splines with four knots at the 5th, 35th, 65th, and 95th centiles were used to examine the association between sleep duration per night and ADL disability. The generalized linear analytic models incorporated a range of covariates, including age, sex, education, marital status, tobacco and alcohol consumption, depression, place of residence, sensory impairment, self-reported health status, life satisfaction, daytime napping, chronic disease status, and sample weights. Multiple imputation with 5 replications and a chained equation approach was adopted to deal with missing data. We also conducted analyses stratified by sex to investigate sex-specific associations. A two-sided *P*<.05 was considered to be statistically significant. All data analyses were performed with Stata version 14.0 (StataCorp).

## Results

### Sample Characteristics

**Figure 1.**
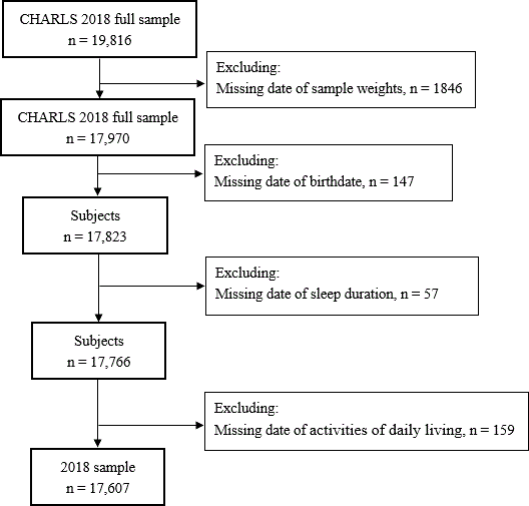
Flowchart of the study sample from the 2018 China Health and Retirement Longitudinal Study (CHARLS).

A schematic flow diagram of the study sample is shown in Figure 1. After filtering, our final study sample included 17,607 people with and without ADL disability.

Table 1 shows the characteristics of the study population in the 2018 wave of the CHARLS. Among the 17,607 participants, 8375 (47.6%) were men and 9232 (52.4%) were women, with a mean (SD) age of 62.7 (10.0) years. A total of 3350 participants (19.0%) reported ADL disability. Compared with people with normal ADL function, individuals with ADL disability were more likely to have extremely shorter (≤4 hours) sleep duration and longer (≥10 hours) sleep duration (all *P*<.001). Participants with ADL disability also tended to have long napping during the day (≥90 minutes). In addition, there were significant differences between ADL disability groups concerning sex, education, tobacco and alcohol use, married status, residence, depressive status, chronic disease conditions, sensory impairments, life satisfaction, and self-reported general health status (all *P*<.001).

**Table 1.** Characteristics of participants by depression status in the 2018 China Health and Retirement Longitudinal Study^a^.

Characteristics	Total sample (N=17,607)	ADL^b^ disability (n=3350)	No ADL disability (n=14,257)	*P* value
Sleep duration per night, hours, mean (SD)	6.2 (2.0)	5.7 (2.5)	6.3 (1.9)	<.001
Sleep duration per night, hours, n (%)				<.001
≤4	3205 (18.2)	1091 (32.6)	2114 (14.8)	
5	2604 (14.8)	493 (14.7)	2111 (14.8)	
6	3847 (21.9)	552 (16.5)	3295 (23.1)	
7	2936 (16.7)	359 (10.7)	2577 (18.1)	
8	3314 (18.8)	450 (13.4)	2864 (20.1)	
9	804 (4.6)	158 (4.7)	646 (4.5)	
≥10	897 (5.1)	247 (7.4)	650 (4.6)	
Age, mean (SD), y	62.7 (10.0)	68.0 (10.2)	61.5 (9.5)	<.001
Sex, n (%)				<.001
Men	8375 (47.6）	1253 (37.4)	7122 (50.0)	
Women	9232 (52.4）	2097 (62.6)	7135 (50.1)	
Education, n (%)				<.001
Illiterate	4176 (23.7)	1250 (37.3)	2926 (20.5)	
Middle school and below	11,321 (64.3)	1913 (57.1)	9408 (66.0)	
High school and above	2110 (12.0)	187 (5.6)	1923 (13.5)	
Tobacco use, n (%)				<.001
Never	10,074 (57.8)	2063 (62.4)	8011 (56.8)	
Current	7349 (42.2)	1243 (37.6)	6106 (43.3)	
Alcohol use, n (%)				<.001
Never	11,442 (65.0)	2479 (74.0)	8963 (62.9)	
Current	6165 (35.0)	871 (26.0)	5294 (37.1)	
Married, n (%)	13,843 (78.6)	2369 (70.7)	11,474 (80.5)	<.001
Residence, n (%)				<.001
Rural	13,997 (79.7)	2812 (84.0)	11,185 (78.7)	
Urban	3567 (20.3)	534 (16.0)	3033 (21.3)	
Depression, n (%)	6182 (35.1)	1859 (55.5)	4323 (30.3)	
Daytime napping, minutes, n (%)				<.001
None	6727 (38.2)	1301 (38.8)	5426 (38.1)	
≤30	1462 (8.3)	279 (8.3)	1183 (8.3)	
31‐90	6839 (38.8)	1204 (35.9)	5635 (39.5)	
≥90	2579 (14.7)	566 (16.9)	2013 (14.1)	
Chronic disease conditions, n (%)				<.001
None	9816 (55.8)	1368 (40.8)	8448 (59.3)	
Mild	6736 (38.3)	1595 (47.6)	5141 (36.1)	
Severe	1055 (6.0)	387 (11.6)	668 (4.7)	
Visual impairment, n (%)	14,535 (82.9)	3029 (91.5)	11,506 (80.9)	<.001
Hearing impairment, n (%)	11,706 (66.6)	2695 (80.7)	9011 (63.3)	<.001
Life satisfaction (satisfied), n (%)	14,365 (81.6)	2267 (67.7)	12,098 (84.9)	<.001
Self-reported general health status (good), n (%)	3846 (21.8)	218 (6.5)	3628 (25.5)	<.001

a Missing data for the following characteristics: tobacco use (184, 1.0%), residence (43, 0.2%), visual impairment (77, 0.4%), hearing impairment (35, 0.2%), life satisfaction (1354, 7.7%), and self-reported general health status (1241, 7.0%).

b ADL: activities of daily living.

### Association Between Sleep Duration and ADL Disability

The association between sleep duration and ADL disability is shown in Table 2. In the model adjusting for age and sex (Model 1), individuals who slept ≤4 hours, 5 hours, 6 hours, 9 hours, and ≥10 hours had a higher risk of ADL disability than the reference group (7 hours per night). After adjusting for age, sex, and other potential confounders (Model 2), the association between 6 hours per night and ADL disability disappeared, while the associations between short (≤4 and 5 hours) and long (9 and ≥10 hours) sleep duration and ADL disability remained the same, with ORs of 1.91 (95% CI 1.60‐2.27; *P*<.001), 1.33 (95% CI 1.09‐1.62; *P*=.006), 1.48 (95% CI 1.13‐1.93; *P*<.001), and 1.88 (95% CI 1.47‐2.41; *P*<.001), respectively.

**Table 2.** Associations between sleep duration and activities of daily living (ADL) disability among participants from the 2018 China Health and Retirement Longitudinal Study.

	OR^a^ (95% CI)	*P* value
Sleep duration per night, hours		
Model 1^b^		
≤4	2.85 (2.48‐3.26)	<.001
5	1.51 (1.30‐1.76)	<.001
6	1.17 (1.01‐1.36)	.031
7	[Reference]	-^c^
8	1.08 (0.92‐1.25)	.342
9	1.41 (1.14‐1.75)	.002
≥10	2.03 (1.68‐2.46)	<.001
Model 2^d^		
≤4	1.91 (1.60‐2.27)	<.001
5	1.33 (1.09‐1.62)	.006
6	1.11 (0.93‐1.34)	.246
7	[Reference]	-^c^
8	1.06 (0.88‐1.27)	.549
9	1.48 (1.13‐1.93)	<.001
≥10	1.88 (1.47‐2.41)	<.001

a OR: odds ratio.

b Model 1 was adjusted for age and sex.

c Not applicable.

d Model 2 was adjusted for age, sex, education, marital status, tobacco use, alcohol use, afternoon napping, residence, depression, chronic disease conditions, visual impairment, hearing impairment, life satisfaction, self-reported health status, and sample weights.

### Sensitivity Analyses

**Figure 2.**
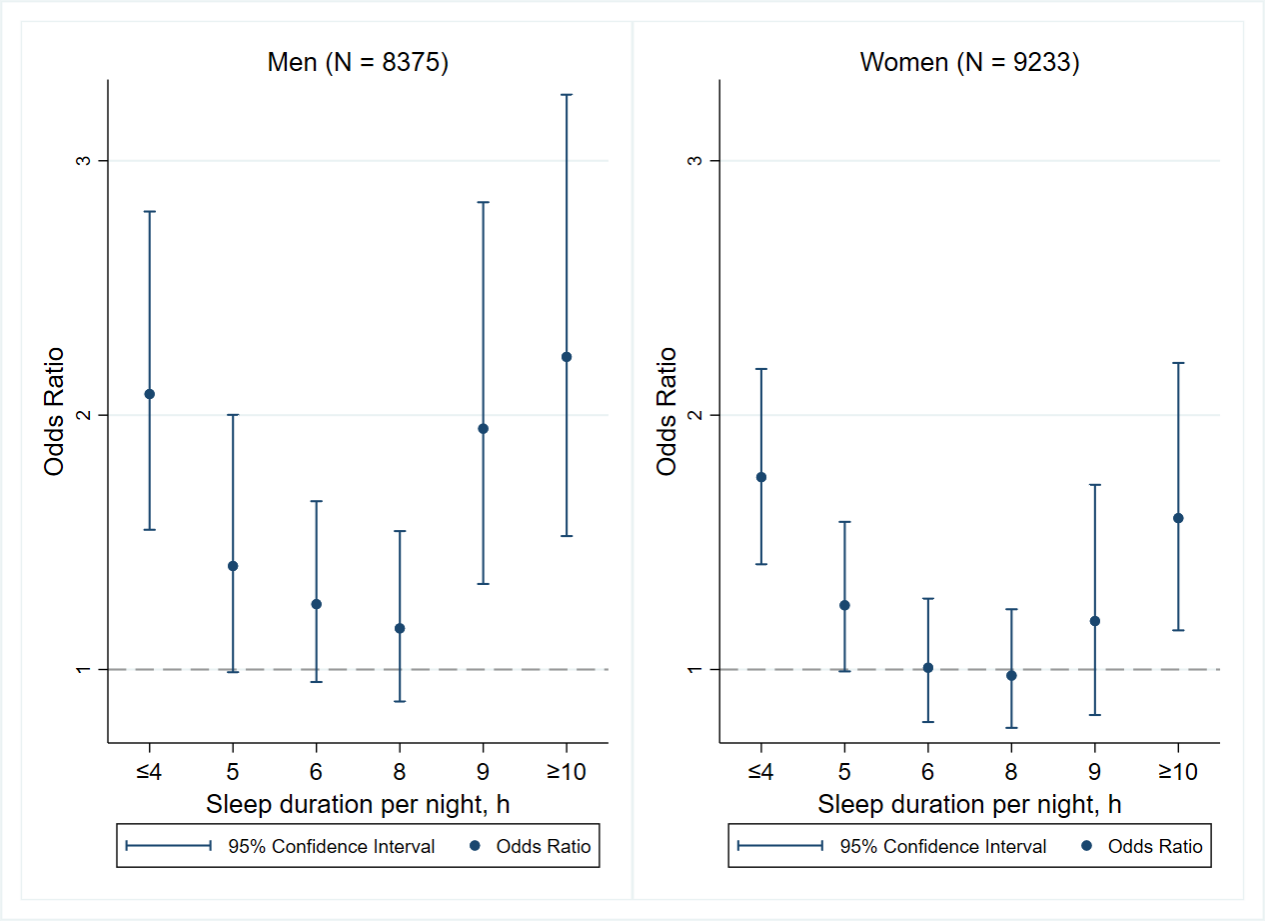
Sex-specific effect of sleep duration on the activity of daily living disability.

Figure 2 provides the results of stratified analysis by sex. Our findings found that for both men and women, short (≤4 hours) and long (9 and ≥10 hours) sleep duration were associated with higher ADL disability compared to the reference group. However, after adjusting for covariates, men with less than 4 hours (OR 2.08, 95% CI 1.55‐2.80), 9 hours (OR 1.95, 95% CI13.4‐2.84), and above 10 hours per night (OR 2.23, 95% CI 1.52‐3.26) had a higher risk than women did.

### Nonlinear Relationship Between Sleep Duration and ADL Disability

**Figure 3.**
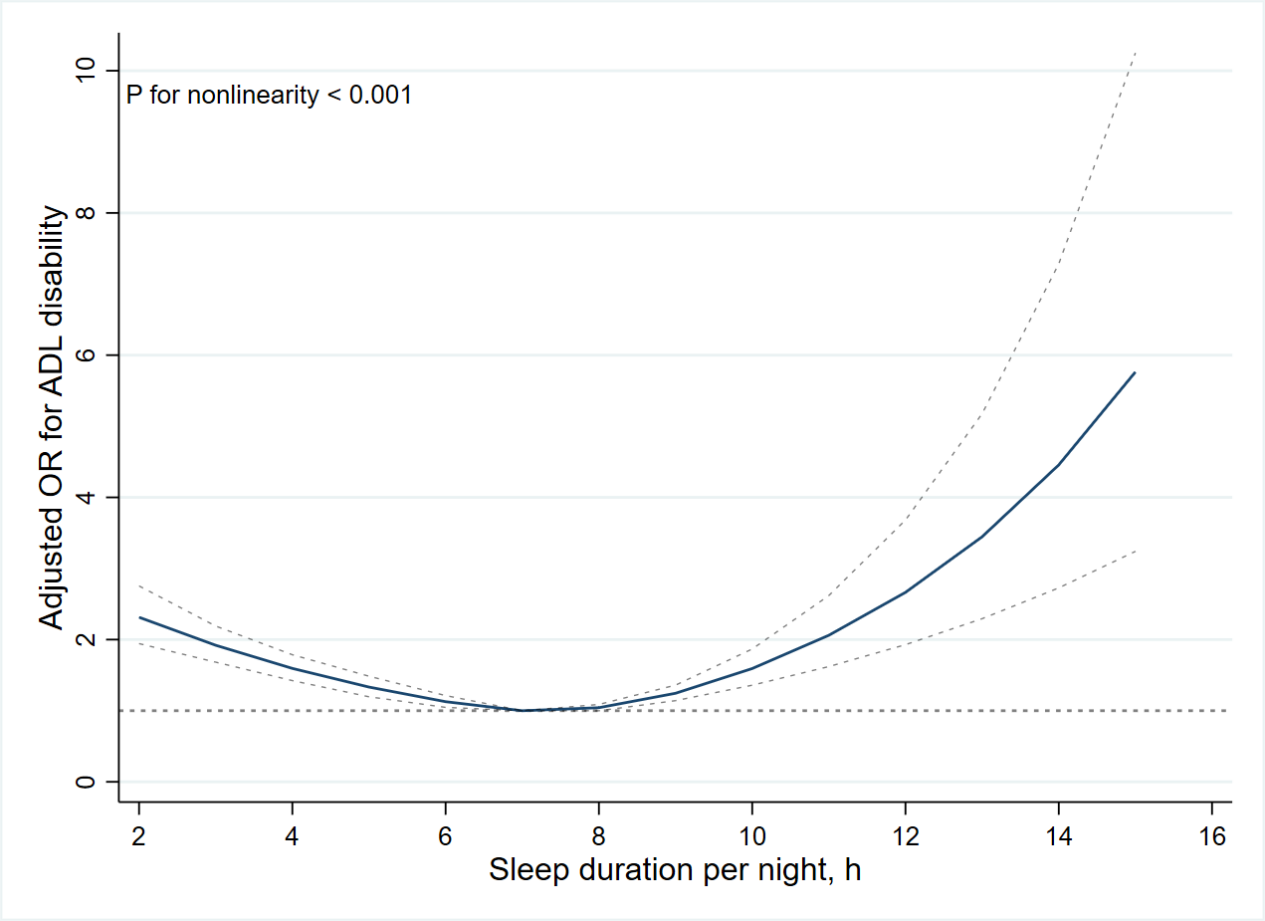
Nonlinear relationship of sleep duration and activity of daily living disability. OR: odds ratio; ADL: activities of daily living.

In Figure 3, restricted cubic splines were used to flexibly model and visualize the relations between predicted sleep duration and ADL disability. Multivariable-adjusted restricted cubic splines analyses suggested U-shaped associations between sleep duration and ADL disability. The risk of ADL disability was negatively correlated with sleep duration until it bottomed out at 7 hours (OR 0.83, 95% CI 0.79‐0.87; *P*<.001). However, when the sleep duration was higher than 7 hours, the risk of ADL disability increased significantly (OR 1.19, 95% CI 1.12‐1.27; *P*<.001).

## Discussion

### Principal Findings and Comparison With Previous Works

In this nationally representative study, using the CHARLS database, a statistically significant U-shaped association was found between sleep duration and ADL disability. Individuals with short and long sleep duration were associated with a higher risk of ADL disability. To the best of our knowledge, the current study stands as one of the most extensive examinations of the correlation between sleep duration and ADL disability in the Chinese population.

Compared to other countries, the prevalence rates of ADL disability among older adults were higher in China [39], with one study observing a rate as high as 41% [40]. In contrast, in America, the percentage of older adults aged 65‐74 years living with functional disability was 25.4% in 2015 [41]. The measurement of ADL disability serves as a crucial tool for monitoring the efficacy of therapeutic interventions across a range of diseases [42]. Given the well-established link between ADL disability and functional ability, it is important to conduct a study on the relationship between sleep duration and ADL disability.

Previous studies have estimated the correlation between sleep duration and ADL disability. The National Health & Aging Trends Study in the United States has demonstrated that insomnia symptoms were correlated with increased likelihood of functional limitations in later life [43]. The Centers for Disease Control and Prevention reported that adults who slept less than 7 hours per night were more prone to difficulties with daily activities than those who slept 7‐9 hours per night [44]. Data from the New Integrated Suburban Seniority Investigation Project in Japan also suggested a heightened risk of incident disability in older adults associated with sleeping less than 6 hours per night [16]. However, this study found no significant link with long sleep duration, potentially due to its limited sample size (n=256) and subject detection bias. Conversely, data from 486,619 stroke survivors from the National Health Interview Survey revealed that long sleepers (≥9 hours) were more likely to experience problems with instrumental activities of daily living compared to average sleepers [13]. Our results are in accordance with the studies highlighting the increased risk of ADL disability associated with both short and long sleep. Meanwhile, our findings also align with a previous CHARLS study [45], which indicates that sleep durations of short (4 hours or less) and long (9 hours or more) predicted incident ADL disability.

Our results of multivariable-adjusted restricted cubic splines analyses also observed a U-shaped association between sleep duration and ADL disability. Our results lent support to the conclusion that adults aged 18‐60 years were recommended to sleep at least 7 hours each night to foster optimal health and well-being, as suggested in previous studies [8,46]. Furthermore, “Healthy China 2030” echoes this advice, advocating for a sleep duration of 7‐8 hours per day for Chinese adults. Insufficient or excessive sleep duration per night was associated with an increased risk for coronary heart disease, cognitive decline, and functional limitations [30,45,47]. The evidence from China Kadoorie Biobank indicated that 23% of Chinese adults reported getting below 6 hours of sleep duration and 16% reported exceeding 10 hours [48]. The observed nonlinear association underscores the ongoing necessity for public education regarding sleep health and opportunities for healthcare providers to engage patients in discussions about the significance of sleep and to promote sleep health initiatives. Future research to prevent functional limitations may consider intervention strategies focused on sleep hygiene and inform the development of health policies.

The mechanisms by which sleep duration influences ADL disability remain unclear. Several plausible explanations have been proposed to explain the correlation between sleep duration and ADL disability. One possible explanation is tied to the pathology of age-related macular degeneration pathology. An exploratory study involving 277 patients with age-related macular degeneration found significant alterations in the expression levels of immediate early response 3 (IER-3), tissue inhibitor of metalloproteinase-3 (TIMP-3), beta 3-glucosyltransferase (B3GALTL), hepatic lipase (LIPC), and HtrA serine peptidase 1 (HTRA1) among individuals with sleep deprivation or patients with age-related macular degeneration who experienced an increase in sleep duration [49]. Meanwhile, physical activity markedly contributed to exacerbating the severity of age-related macular degeneration. Furthermore, IER-3 and TIMP-3 also had a role in regulating mechanisms in circadian rhythms [49]. The second explanation links the relationship between sleep duration and ADL disability to psychological distress [50]. Similarly, sleep duration also affected the relationship between ADL disability and psychological distress as a mediating pathway, which might also affect ADL disability by causing psychological distress [6]. However, the underlying mechanism governing the association between sleep duration and ADL disability remained uncertain.

In addition, our stratified analysis revealed an elevated risk of ADL disability among men with short and long sleep durations. Sex differences in sleep duration cannot be ignored, as men generally tend to sleep less than women throughout their lifespan [51]. Our findings align with those from the Swedish panel study of living conditions among the oldest old, which showed stronger associations between ADL and instrumental activities of daily living as well as other health indicators among men compared to women between 1992 and 2002 [52]. A longitudinal observation study of 3609 adults aged 65‐89 years from the Korean National Health and Nutrition Examination Survey also found that men had 2‐3 times higher odds of developing ADL disability than women [53]. One plausible explanation for this sex difference could be the more severe impact of conditions such as stroke, cancer, and pulmonary disease on older men compared to women, potentially due to men’s greater propensity for alcohol and tobacco consumption, thereby increasing the greater risk of ADL disability.

### Strengths and Limitations

Our study boasts significant strengths, notably its utilization of a nationally representative sample of Chinese adults aged 45 years and older, rather than relying on clinical samples or occupational cohorts. Furthermore, to our knowledge, it stands as one of the pioneering population-based studies to incorporate both sleep duration and ADL disability. However, several limitations of the current study merit consideration. First, due to its cross-sectional design, we are unable to establish causal relationships between sleep duration and ADL disability based on the observed associations, necessitating a cautious interpretation of the findings. Second, sleep duration was solely assessed through self-reporting, which may introduce potential biases. Third, the CHARLS questionnaire lacks comprehensive sleep-related information, such as objective assessments of the sleep quality, sleep disturbance, and difficulties initiating sleep, which could potentially act as a confounding factor, influencing our estimates. Finally, the binary classification of ADL disability, rather than a continuous scale, might underestimate the associations between sleep duration and ADL disability. Additionally, despite controlling for a range of covariates in the analysis, we cannot discount the influence of unknown factors in this study.

### Conclusions

A nationally representative sample in China revealed a statistically significant U-shaped relationship between sleep duration and ADL disability. The U-shaped pattern underscores the importance of monitoring ADL disability in middle-aged and older individuals who exhibit either insufficient or excessive sleep duration. It is imperative for future longitudinal studies to elucidate the causal or bidirectional links between sleep duration and ADL disability, while also delving into the potential mechanisms underlying this intricate interplay.

## Abbreviations

ADL: activities of daily living
B3GALTL: beta 3-glucosyltransferase
CHARLS: China Health and Retirement Longitudinal Study
HTRA1: HtrA serine peptidase 1
IER-3: immediate early response 3
LIPC: hepatic lipase
OR: odds ratio
TIMP-3: tissue inhibitor of metalloproteinase-3
WHO: World Health Organization
